# Analyzing the popularity of YouTube videos that violate mountain gorilla tourism regulations

**DOI:** 10.1371/journal.pone.0232085

**Published:** 2020-05-21

**Authors:** Ryoma Otsuka, Gen Yamakoshi

**Affiliations:** Graduate School of Asian and African Area Studies, Kyoto University, Kyoto, Japan; The University of Hong Kong, CHINA

## Abstract

Although ecotourism is expected to be compatible with conservation, it often imposes negative effects on wildlife. The ecotourism of endangered mountain gorillas has attracted many tourists and functioned as a key component of their conservation. There might be expectations on the part of tourists to observe or interact with gorillas in close proximity and such expectations may have been engendered by the contents of social media in this Information Age. However, the risk of disease transmission between humans and gorillas is a large concern and it is important to maintain a certain distance while observing gorillas to minimize risk. We conducted a content analysis and described the general characteristics of 282 YouTube videos related to mountain gorilla tourism. Humans and gorillas were observed simultaneously in 70% of the videos, and physical contact or close proximity within arm’s reach were identified in 40%. To explore the factors affecting the number of views and likes that these videos received, we ran generalized linear mixed models and performed AIC model selection with 206 videos in which humans and gorillas were observed simultaneously. Videos obtained more views and likes when the thumbnail photos included humans and gorillas together, while videos with thumbnail photos of only gorillas did not obtain more views and likes compared with those that included no gorillas. Moreover, videos obtained more views and likes in cases where physical contact or close proximity within arm’s reach with gorillas were clearly observed, compared with those that did not clearly include close human-gorilla interaction. These results suggest that human-gorilla interaction and proximity with gorillas attract more public attention than gorillas shown by themselves. Our study highlights the importance of further investigation on the direct link between such contents that violate tourism regulations and the conflicting situation.

## Introduction

Ecotourism has become a popular leisure activity and has generated huge tourism revenue [1, 2]. Although ecotourism seems to have many definitions [3], it is generally regarded as an activity that is sustainable, compatible with conservation, generates revenue for conservation, contributes to local and national development, and provides educational opportunities for visitors [e.g. 3, 4]. A recent study estimated that there were 8 billion visits to protected areas in the world per year, generating US $600 billion per year in direct in-country expenditure [2]. Moreover, a previous study demonstrated that the conservation of endangered mammal species is dependent on tourism revenue [5] and tourism can have positive effects on the survival of some endangered species [6].

However, there is also much evidence to suggest that ecotourism in protected areas often has negative effects on wildlife or natural environments [e.g. 7, 8]. These negative effects include long-term behavioral changes driven by human presence [e.g. 4], stress caused by habituation and observation [e.g. 4, 9], and the transmission of human diseases to vulnerable populations such as the great apes [10], among others. Careful and effective control and management of ecotourism is required [11] and it is, therefore, crucial to accurately understand and minimize the negative effects of ecotourism on resources in order to maintain sustainable tourism.

Mountain gorillas (*Gorilla beringei beringei*) were classified as a critically endangered species, but were recently downgraded to an endangered species according to the IUCN Red List [12], with a total population of 1,063 remaining in only two isolated habitats [13]. The Virunga gorilla population is found in the Virunga massif that includes the following three national parks; the Virunga National Park in the Democratic Republic of Congo, the Volcanoes National Park in Rwanda, and the Mgahinga Gorilla National Park in Uganda. The Bwindi gorilla population is found in the Bwindi Impenetrable National Park (hereafter Bwindi) in Uganda. These mountain gorilla habitats are mainly afro-montane forests and are surrounded by agricultural areas with very high human population density [14, 15]. Thus, there had been conflicts between the parks (or protected areas) and surrounding communities [e.g. 16], however, ecotourism has been introduced and functions as a key component of the conservation of mountain gorillas, generating a huge amount of tourism revenue for both their conservation and development of local communities [e.g. 17−19]. Integrated Conservation and Development Projects (ICDPs) implemented around mountain gorilla habitats include various projects such as agricultural support, infrastructure development, and tourism revenue sharing projects [20, 21]. These projects have improved community people’s perceptions of the parks [20, 21], although tourism revenue sharing projects seemingly have challenges associated with structural and political constraints, conflicting interests among different organizations and corruption [18, 19]. Sandbrook [22] demonstrated that, in Bwindi, the amount of tourism revenue greatly exceeded the amount of revenue from non-tourism sources of income to this area, even though a significant amount of tourism revenue leaked outside of this area. Moreover, the population of mountain gorillas has continued to increase for more than three decades in the Virunga massif and for perhaps more than two decades in Bwindi [e.g. 12, 13, 23, 24] and there is evidence that suggests that ecotourism has contributed to this trend. The study by Robbins et al. [25] showed the positive effects of ecotourism in mountain gorilla conservation by demonstrating that human interventions such as veterinary treatments and the daily monitoring of each habituated gorilla group for tourism and research contributed to an increase in the population (in particular to the higher population growth rate in habituated gorilla group compared to unhabituated group).

In mountain gorilla tourism, however, the risk of disease transmission between humans and gorillas has been the greatest concern [e.g. 10, 26] as in other great ape tourism [e.g. 27, 28]. A previous study showed that tourists visiting Kibale National Park, Uganda, had a higher prevalence of diseases [29], indicating that the tourists who participate in mountain gorilla tourism may have a higher prevalence of diseases as well. In the Virunga gorilla population, respiratory disease was the second greatest cause of mortality after physical trauma [30]. There have been outbreaks caused by human pathogens such as fatal respiratory virus infections [31] and the frequency of respiratory disease outbreaks is increasing [32].

The international guidelines for mountain gorilla tourism were set out and have been updated to minimize the risk of disease transmission and stressful interaction between humans and gorillas [26]. These guidelines include the regulation of the maximum number of tourists (up to eight tourists) per habituated gorilla group per day, the limit of a maximum of one tourist visit per day, the limitation of observation hours (maximum 1 hour), the use of surgical masks during observation, and the minimum distance rule called the 7 m rule [26]. Most of these regulations have been implemented in the four national parks, while the decision to use masks was postponed and not implemented except in the Virunga National Park in the Democratic Republic of Congo [26]. The 7 m rule is a regulation in which tourists and rangers should always maintain a distance of at least 7 m from the gorillas in order to prevent droplet infection [26]. However, previous studies have shown that the 7 m rule may not be strictly observed, based on self-reported information of tourists who tracked gorillas in Bwindi [33, 34]. There are several studies suggesting that the degree of proximity to wild animals is one of the main factors that affects tourists’ satisfaction [e.g. 35, 36]. It is likely that proximity to the mountain gorillas also increases the level of the tourists’ satisfaction. However, in great ape tourism, the desires or expectations of tourists to be in closer proximity to wildlife is not compatible with conservation. Although this conflicting situation may undermine mountain gorilla conservation, tourists’ expectations for closer proximity to mountain gorillas has not yet been investigated.

Tourists’ expectations of close experiences with mountain gorillas may be created by tourism guidebooks, websites, TV programs, news, and word of mouth. Recently, many tourism studies have focused on social media and showed that social media has been playing an important role in tourism, especially with regard to information search [37] and experience sharing [38]. These studies suggest that tourists’ expectations may often be created by contents on social media in this Information Age [e.g. 37, 38]. Moreover, social media has increasingly been used in conservation science to analyze human-nature interactions and contribute to biodiversity conservation [39, 40]. The use of social media is part of a broader, emerging approach in conservation science that tries to understand human-nature interactions using an enormous digital data set. For instance, Ladle et al. 2016 [41] described the potential of analyzing changes in word frequencies in an enormous digital text database in order to better understand complex interactions between human cultures and the nature. It is generally thought that a deeper understanding of such interactions has the potential to contribute to the practice and science of conservation [41]. Various online platforms including social media have also been used in order to identify emerging issues that may affect global conservation and biological diversity but are not yet recognized well [e.g. 42]. Among multiple sources of digital data, social media data has been used as a powerful tool for conservation in particular by analyzing the main contents (such as text, photos, and videos), user profiles, geo-tags, time-stamps, and social engagement (such as views, likes, comments) [39, 40, 43]. For instance, Hausmann et al. 2018 [43] demonstrated that social media has the potential to be utilized to remotely and cost-effectively understand tourists’ preferences or expectations in wildlife tourism. Di Minin et al. 2019 [44] provided a framework to investigate illegal wildlife trade using social media data and machine learning.

In the present study, we focused on YouTube, a freely accessible internet social media [45]. YouTube is the world’s second-largest search engine and the third-most visited site after Google and Facebook [46]. There are several studies that used this platform for conservation. For instance, the study by Nekaris et al. [47] focused on YouTube videos in which slow lorises were tickled and demonstrated how misleading contents on YouTube can negatively affect public perception of wild animals and/or their conservation. In addition, the data from this platform was used to look at illegal sports hunting in Brazil [48] and recreational fisheries (angling and spearfishing) [49]. In both studies, the authors mainly analyzed the contents of videos and social engagement such as the number of views likes, dislikes, and comments [48, 49]. These studies suggested that this platform has the potential to be utilized in order to better understand the human-nature interactions through analyzing the video contents related to recreational activities and social engagement by viewers [39, 40, 43, 48, 49]. As mentioned earlier, there is a high possibility that potential tourists who are interested in mountain gorilla tourism will watch photos or videos on social media. Indeed, some tourists claimed that they watched videos relating to mountain gorilla tourism on YouTube and other social media before they visited Bwindi (personal observation). However, no study has explored the potential effects of YouTube videos related to mountain gorilla tourism on potential tourists’ expectations. The aim of this paper is to contribute to the overall understanding of the potential effects of these contents related to the ecotourism of the endangered species. In this paper, we first describe the general characteristics of these YouTube videos. Thereafter, we investigated the factors that affect the number of views and likes to better understand what features of the videos affect social attention and evaluations, focusing particularly on human-gorilla interaction, namely proximity, during the tourism experience.

## Materials and methods

### Ethical statement

The use of YouTube contents in this study was approved by the Ethics Review Committee of the Center for African Area Studies, Kyoto University (Application No.: 18-04B). We only used videos that were freely available to the public. Our methodology complies with YouTube’s terms of service.

### Video selection and information recording

We searched YouTube videos related to mountain gorilla tourism using the search feature on the YouTube website on 28 April 2019. We used the keywords “gorilla trekking” and “gorilla tracking”. Mountain gorilla tourism is generally called and advertised as either of the two phrases. After excluding videos which had no relation to gorillas, a total of 316 videos were identified and added to a list on the website. However, nine videos were related to only western lowland gorilla tourism, five to eastern lowland gorilla tourism, and one focused only on orphan mountain gorillas and their interactions with staff members in the orphan center. Furthermore, the population of mountain gorillas (whether Bwindi or Virunga) were not identified in eleven videos and seven did not include gorillas but only humans explaining mountain gorilla tourism. These were, therefore, excluded and a total of 282 videos were used for the following coding process.

The title, uploader, web address, date of upload, and length of each video was recorded. We calculated the days after upload by subtracting the date of upload from April 28, 2019. The number of subscribers of the channel was also recorded. As an index for degree of exposure and viewers’ engagement, the number of views and likes were recorded. Generally, the number of views increases when the video page is opened on YouTube, and the number of likes increases when the like button is pressed during or after watching the video. We also recorded the number of dislikes and comments, however, we did not use these indicators in our analysis because many of our samples had zero dislikes or comments (or they were not available). (please see S1 Dataset).

### Coding

From 29 April to 4 May 2019, RO watched and coded each video systematically. The videos were coded as follows: the population of mountain gorilla (Bwindi/Virunga), the national park (Bwindi/Mgahinga/Virunga/Volcanoes), the presence of silverback gorillas (present/absent), the presence of infant gorillas (present/absent), the use of masks (used/not used/NA), explanation of disease transmission risks (explained/not explained/NA). Although English was used in most videos, other languages were also used in some videos. In these cases, we judged the explanations of disease transmission as unavailable (NA). RO had experience in observing mountain gorillas in Bwindi for more than 10 months and visited Volcanoes National Park as a tourist, thus we were able to reliably categorize the gorillas into the two populations. Landscapes and the rangers’ uniforms were also used to code the populations and identify the parks.

In addition, the sampled videos were coded based on the thumbnail photo type. If the thumbnail photos included both humans and gorillas, we coded the videos as “humans and gorillas”. “Human” here refers to anyone who tracked gorillas and mainly refers to tourists but may include park rangers. If the thumbnail included only gorillas, we coded the videos as “gorillas only” and if the thumbnail photos did not include gorillas, we coded the videos as “others”. We also coded the videos into five categories based on the minimum distance between humans and gorillas because it was difficult to estimate the distance accurately as numerical values. If physical contact was clearly observed, the videos were coded as “0 m”. If the minimum distance between humans and gorillas was within arm’s reach, the videos were coded as “AR”. When the minimum distance was more than an arm’s reach between the humans and gorillas, but clearly less than 7 m, we coded the videos as “< 7 m”. If the minimum distance was not clearly less than 7 m or the minimum distance was clearly more than 7 m, we coded the videos as “> 7 m”. When humans and gorillas were not seen simultaneously in the videos, we coded the videos as “NA”. Even if it seemed that gorillas were very close to the lens of the camera, we coded videos as “NA” by following the coding guideline above. The dataset used in the analyses is available in S1 Dataset.

### Data analyses

All analyses in this study were performed using R 3.5.0 [50]. To describe the general characteristics of the sampled YouTube videos, we calculated the frequency, percentage, mean (SD), and median (IQR). To test the coding reliability for two coding areas that seemed to be relatively difficult to judge, namely the thumbnail photos and the minimum distance, 100 videos were randomly selected from the 282 samples and were independently coded by another coder. Following Keelan et al. [51], we calculated the weighted *k* (Cohen’s Kappa) statistic using the kappa2 function in the irr package. The weighted *k* statistic for agreement between the two coders on the thumbnail photo type and the minimum distance were 0.90 and 0.85 respectively, which are generally regarded as highly reliable [52]. Therefore, we used the codes by RO for the thumbnail photo type and the minimum distance.

To explore the factors affecting the number of views and likes of the sampled YouTube videos, we ran generalized linear mixed models with Poisson error distribution and log link function using the glmer function in the lme4 package [53, 54]. The response variables (i.e. the number of views and likes) were count data with overdispersion. In order to consider overdispersion, we included ID of each video as random intercept effects in the models. We used bobyqa as an optimizer. Prior to the analysis, we excluded the 63 videos whose minimum distances were not estimated and coded as “NA”. We also excluded five videos whose main content was mating scenes of mountain gorillas because some of these mating videos obtained exceptional views, likes, and dislikes. Thereafter, eight videos were excluded because the number of subscribers was not available. Therefore, we used a total of 206 videos in the modeling. For both response variables, the explanatory variables were the number of subscribers, the days after upload, the length of the video (in seconds), the population (Bwindi or Virunga), the presence of silverback gorillas (presence/absence), the presence of infant gorillas (presence/absence), the thumbnail photo type (humans and gorillas/gorillas only/others), and the minimum distance (0 m/AR/< 7 m/> 7 m). Please see the supplementary information for details of the variables in the modeling (S1 Table). We tested the multicollinearity of the explanatory variables using the vif function in the car package. The variance inflation factors were smaller than 2. Therefore, we assessed that the multicollinearity did not affect the results. The continuous variables were centered and standardized by subtracting the mean value and then dividing by the SD so that their estimates are comparable in magnitude within models [55]. We ran a set of models with all possible combinations of the explanatory variables (known as all-subset approach) and ranked them based on Akaike’s information criterion (AIC) values [56]. In AIC model selection, although Δ AIC less than 2 cut-off rule have been widely applied in previous studies [e.g. 57], there are still some debates on when models should be dismissed [57–59]. In this study, we selected all models with Δ AIC value less than 6, which is recommended by simulation study in order to have approximately a 95% chance of including the truly most parsimonious model in the candidate set [59]. In order to avoid getting overly complex models, we considered whether candidate models were “nested” or not [e.g. 57, 59]. Nested models are more complex models (i.e. having additional explanatory variable(s)) compared to any model that had a lower AIC value [e.g. 57, 59] and the simulation study demonstrated that removing nested models from the candidate set does not necessarily affect the chance of including the truly most parsimonious models [59]. Therefore, in the present study, we first presented all candidate models (with Δ AIC value less than 6) with their model structure, log-likelihood, df, AIC, Δ AIC, and Akaike weight. Then, we presented the incidence rate ratio (IRR) (i.e. the exponent of estimate) and 95% confidence interval (CI) of each explanatory variable of the first ranked model and “non-nested” models in the candidate set in the result. We used the confint function to calculate the 95% CI of estimate of each explanatory variable and we exponentiated the calculated values to obtain the 95% CI of incidence rate ratio (IRR) of each explanatory variable. We also presented the summary of the full models as (S2 & S3 Tables).

## Results

### The characteristics of the sampled YouTube videos

**Fig 1:**
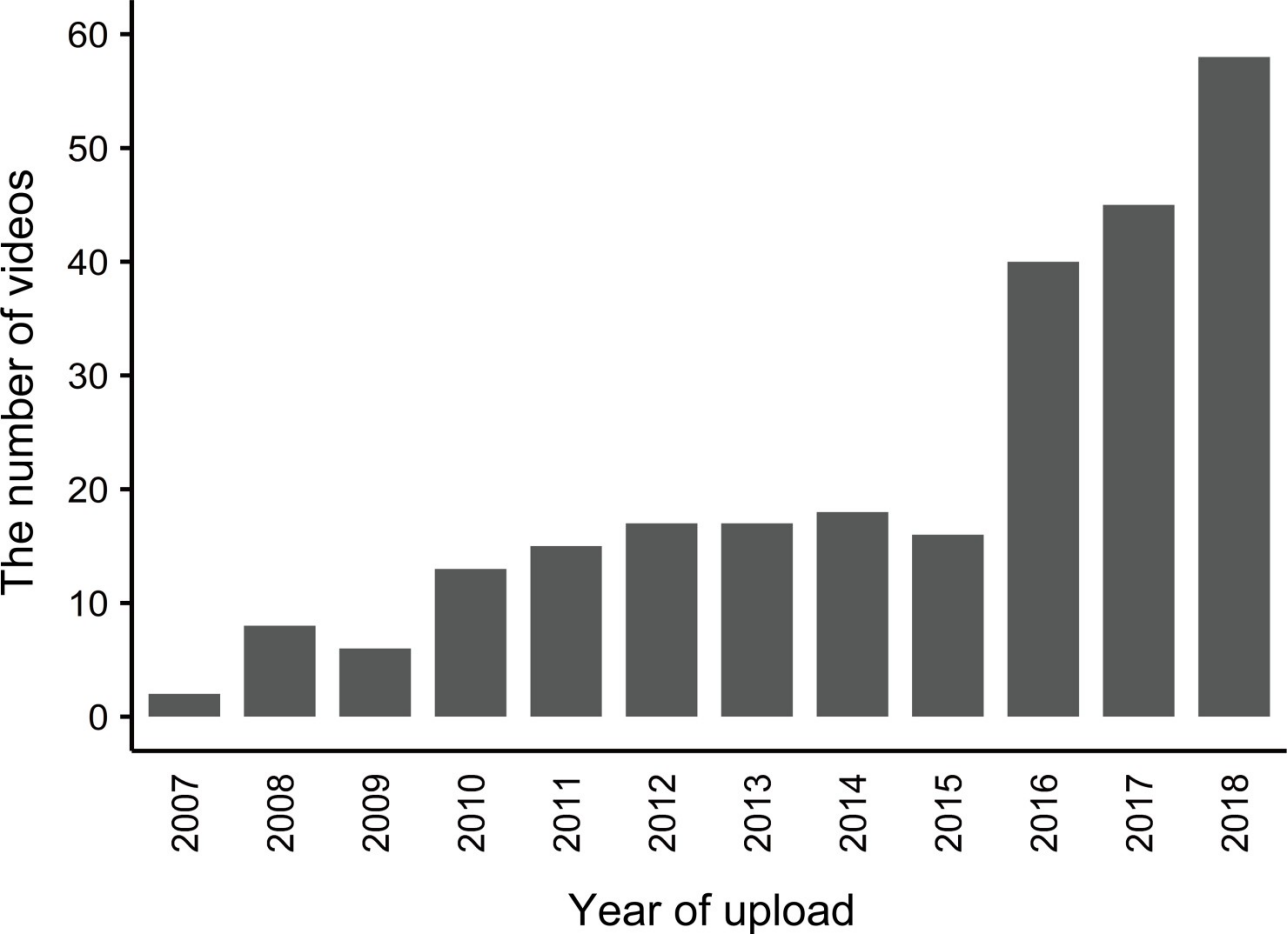
The number of YouTube videos related to mountain gorilla tourism that were uploaded in each year between 2007 and 2018.

The sampled 282 YouTube videos were uploaded by 265 unique uploaders and the mean (SD) number of videos per uploader was 1.07 (0.32). The number of videos had roughly been increasing since 2007 until 2015, but has drastically increased since 2016 (Fig 1). The annual mean (SD) number of videos uploaded between 2007 and 2015 was 12.4 (5.7) and 47.7 (9.3) between 2016 and 2018. The number of videos uploaded from 1 January 2019 until 29 April 2019 (about four months) was 27, which was already 46.6% of the number of videos uploaded in 2018. The median (IQR) days after upload was 988.5 (1794.6) days and the median (IQR) length of the videos was 274.5 (413.3) seconds. The median (IQR) number of subscribers was 105.0 (1120.0) although the information for 12 videos were not available. The median (IQR) number of views were 1,272.0 (6,777.0). The median (IQR) number of likes were 8.0 (32.5), although the information for 2 videos were not available. The maximum number of views and likes were 8,160,553 and 17,000 respectively. The total number of views and likes of the sampled 282 videos were 26,373,615 and 67,157, respectively.

**Fig 2:**
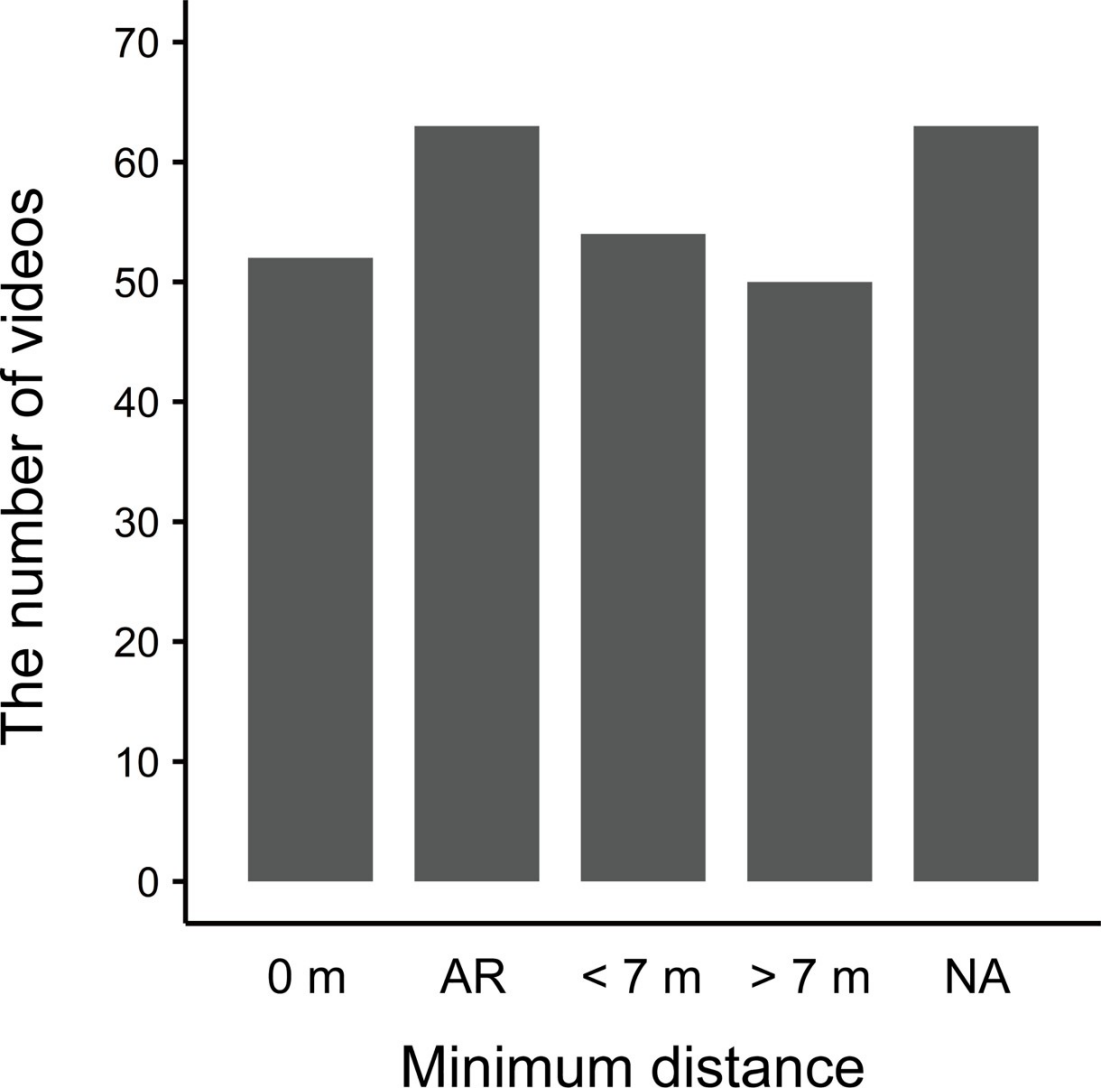
The number of YouTube videos coded for each minimum distance categories. The category ‘AR’ means close proximity within arm’s reach.

As for the population, 146 (51.8%) videos were categorized as the Bwindi gorilla population and 136 (48.2%) as the Virunga gorilla population. Among the videos containing the Virunga population gorillas, eight were recorded in Mgahinga Gorilla National Park, 17 in Virunga National Park, and 111 in Volcanoes National Park. The use of masks was identified in only 10 (3.5%) videos, while 62 (22.0%) videos were coded as “NA” and 210 (74.5%) videos were coded as “not used”. The risk of disease transmission was explained in only eight (2.5%) videos, while 29 (10.3%) videos were coded as “NA” and 246 (87.2%) videos were coded as “not explained”. As for thumbnail photo type, 43 (15.2%) videos included humans and gorillas in the thumbnail photos, 191 (67.7%) included only gorillas, and 48 (17.0%) did not include gorillas and were thus coded as “others”. As for the minimum distance between the humans and gorillas, physical contact with gorillas (0 m) was observed in 52 (18.4%) videos and close interaction within arm’s reach (AR) was observed in 63 (22.3%) videos. Thereafter, 54 (19.1%) videos were coded as “< 7 m,” 50 (17.7%) as “> 7 m,” and 63 (22.3%) as “NA” (Fig 2).

### Factors affecting the number of views

**Fig 3:**
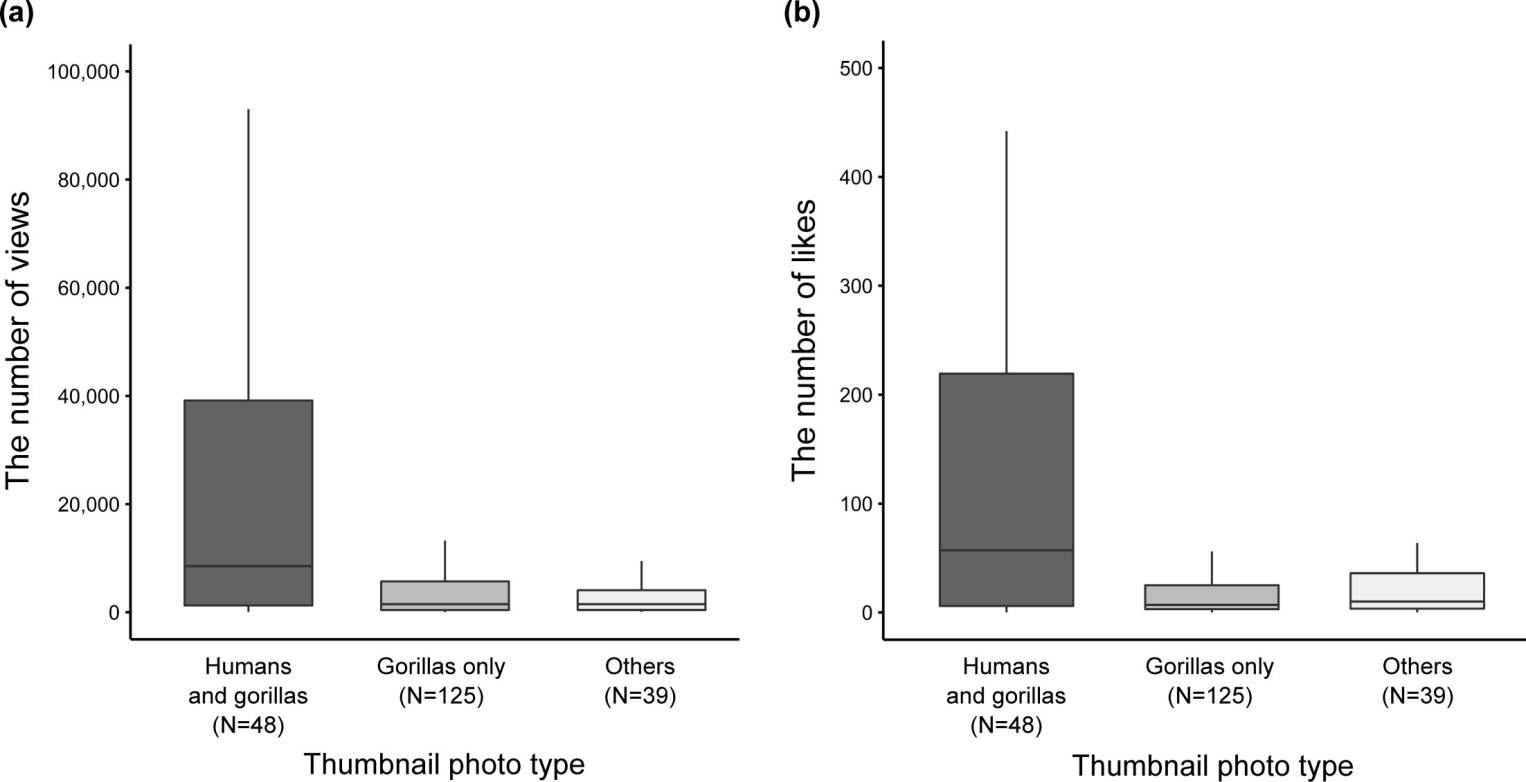
A comparison of the number of views and likes of the sampled YouTube videos related to mountain gorilla tourism (N = 206) between three different thumbnail photo types (a, b). The upper and lower hinges of boxes represent the first and third quartiles and the thick lines in boxes show the medians. The upper whiskers extend out from boxes to the largest value no further than 1.5 * IQR from the hinges while the lower whiskers extend from the boxes to the smallest value no further than 1.5 * IQR of the hinges. As for the y-axis of each figure, only the parts under 100,000 (a) and 500 (b). AR means close proximity within arm’s reach.

**Fig 4:**
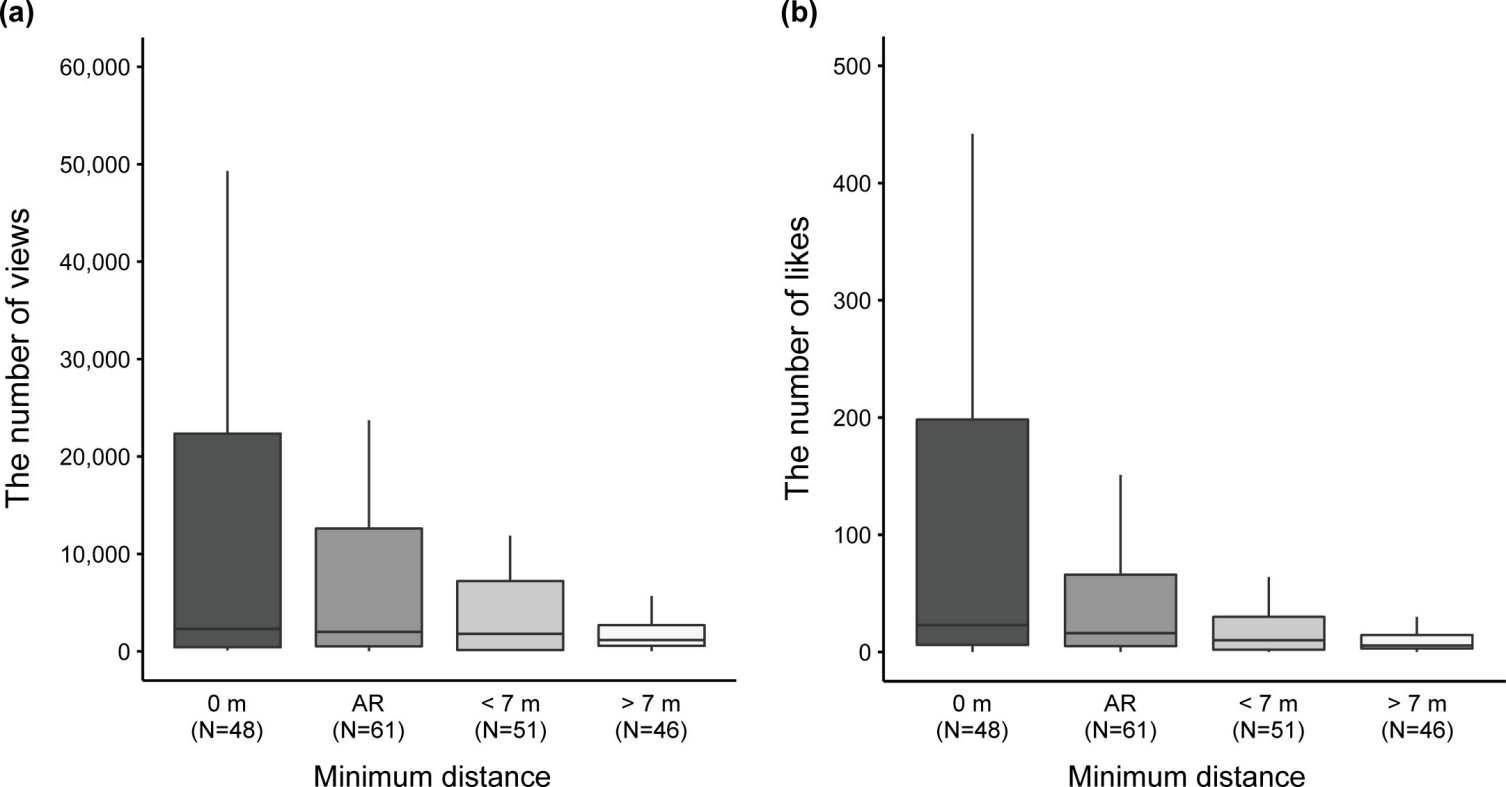
A comparison of the number of views and likes of the sampled YouTube videos related to mountain gorilla tourism (N = 206) between four different minimum distance categories (a, b). The upper and lower hinges of boxes represent the first and third quartiles and the thick lines in boxes show the medians. The upper whiskers extend out from boxes to the largest value no further than 1.5 * IQR from the hinges while the lower whiskers extend from the boxes to the smallest value no further than 1.5 * IQR of the hinges. As for the y-axis of each figure, only the parts under 60,000 (a), and 500 (b) are shown. AR means close proximity within arm’s reach.

In the results of the modeling and the AIC model selection exploring the factors that affect the number of views of the videos (N = 206), we obtained a set of 20 candidate models (with Δ AIC values less than 6) out of 255 possible models (Table 1). Among 19 models except for the first ranked model, three models were non-nested, (i.e. other 16 models were more complex versions of those with lower AIC values) (Table 1). The first ranked model with the minimum AIC value included five explanatory variables, including the days after upload, the number of subscribers, the presence of silverback gorillas, the thumbnail photo type, and the minimum distance (Table 1). The number of subscribers and the days after upload and were included in all 20 candidate models and had a positive effect on the number of views (Tables 1 & 2). The length of video, the population (Virunga compared to Bwindi), and the presence of infant were included only in eight candidate models respectively and they were not included in non-nested models (Table 1). Combined with the incidence rate ratio (IRR) (i.e. the exponents of estimates) and 95% CI of these variables in the full model (S2 Table), we considered that the length of video, the population, and the presence of infant gorillas had almost no clear effect on the number of views. The presence of silverback gorillas was included in 11 candidate models and one non-nested model (Table 1). The IRR of the variable implied a positive effect on the number of views, but the 95% CI lower was slightly less than 1.00 in the first ranked model and the ninth ranked non-nested model (Table 2). The thumbnail photo type and the minimum distance had a greater effect on the number of views (Tables 1 & 2). The thumbnail photo type was included in all 20 candidate models (Table 1). Although the 95% CI lower was slightly less than 1.00 in the second ranked non-nested model, those of the first ranked model and the other two non-nested models were more than 1.00 (Table 2). The videos with thumbnail photos that included both humans and gorillas tended to obtain more views than those that did not include gorillas (Table 2, Fig 3A). The IRR of thumbnail photos that included only gorillas were less than 1.00 in the first ranked model and the three non-nested models, but judging from the 95% CI, this variable had almost no clear effect on the number of views compared to those that did not include gorillas (Table 2, Fig 3A). The minimum distance was included in 15 candidate models especially in higher ranked models (with Δ AIC values less than around 3 to 4) and in one non-nested model (Table 1). Although the IRR of “< 7 m” showed a positive effect, the 95% CI included 1.00 in the first ranked model and the second ranked non-nested model (Table 2). On the other hand, a much shorter minimum distance, namely “0 m” and “AR” had positive effects on the number of views compared to the videos coded as “> 7 m” in the first ranked model and the second ranked non-nested model (Table 2, Fig 4A).

**Table 1:** Top-ranked candidate models exploring the factors that affect the number of views of YouTube videos related to mountain gorilla tourism (only the models with Δ AIC values less than 6 are shown). (N = 206).

Rank^a^	Nesting^b^	Explanatory variables								logLik	df	AIC	Δ AIC	Akaike weight
		Number of subscribes	Days after upload	Length of videos	Population	Silverback	Infant	Thumbnail	Minimum distance					
1	-	+	+			+		+	+	-1999.85	10	4019.71	0.00	0.20
2	Non-nested	+	+					+	+	-2001.47	9	4020.94	1.23	0.11
3	Nested	+	+			+	+	+	+	-1999.74	11	4021.48	1.77	0.08
4	Nested	+	+	+		+		+	+	-1999.79	11	4021.58	1.87	0.08
5	Nested	+	+		+	+		+	+	-1999.84	11	4021.69	1.98	0.07
6	Nested	+	+	+				+	+	-2001.31	10	4022.62	2.91	0.05
7	Nested	+	+				+	+	+	-2001.46	10	4022.93	3.22	0.04
8	Nested	+	+		+			+	+	-2001.47	10	4022.94	3.23	0.04
9	Non-nested	+	+			+		+		-2004.51	7	4023.01	3.30	0.04
10	Nested	+	+	+		+	+	+	+	-1999.67	12	4023.33	3.62	0.03
11	Nested	+	+		+	+	+	+	+	-1999.72	12	4023.45	3.74	0.03
12	Nested	+	+	+	+	+		+	+	-1999.78	12	4023.56	3.85	0.03
13	Non-nested	+	+					+		-2006.20	6	4024.40	4.69	0.02
14	Nested	+	+		+	+		+		-2004.29	8	4024.58	4.87	0.02
15	Nested	+	+	+			+	+	+	-2001.30	11	4024.60	4.89	0.02
16	Nested	+	+	+	+			+	+	-2001.31	11	4024.62	4.91	0.02
17	Nested	+	+	+		+		+		-2004.32	8	4024.64	4.93	0.02
18	Nested	+	+			+	+	+		-2004.45	8	4024.89	5.19	0.01
19	Nested	+	+		+		+	+	+	-2001.46	11	4024.93	5.22	0.01
20	Nested	+	+	+	+	+	+	+	+	-1999.65	13	4025.30	5.59	0.01

^a^ Rank based on Akaike information criterion (AIC) values

^b^ "Nested" indicate that the model is a more complex version of a model that has a lower AIC value

**Table 2:** Summary of the models exploring the factors that affect the number of views and likes of YouTube videos related to mountain gorilla tourism (N = 206). The incidence rate ratio (IRR) and 95% confidence interval (CI) of each explanatory variable in the first ranked model and non-nested models are shown.

Model	The first ranked model	The 2nd ranked non-nested model	The 9th ranked non-nested model	The 13th ranked non-nested model
Explanatory variables	Incidence rate ratio (IRR) (95% CI)			
Intercept	529.85 (178.67–1571.14)	1104.12 (526.51–2315.11)	868.88 (315.48–2392.83)	1863.84 (979.11–3446.13)
Number of subscribes	1.77 (1.35–2.31)	1.79 (1.37–2.34)	1.81 (1.38–2.38)	1.83 (1.40–2.41)
Days after upload	2.77 (2.10–3.64)	2.86 (2.17–3.76)	2.54 (1.93–3.35)	2.63 (2.00–3.46)
Length of video	-	-	-	-
Population: Virunga	-	-	-	-
Silverback: Present	2.21 (0.93–5.27)	-	2.29 (0.95–5.52)	-
Infant: Present	-	-	-	-
Thumbnail: Humans and gorillas	2.61 (1.05–6.50)	2.43 (0.97–6.07)	4.17 (1.75–9.90)	3.92 (1.64–9.35)
Thumbnail: Gorillas only	0.65 (0.32–1.33)	0.65 (0.32–1.34)	0.77 (0.38–1.58)	0.78 (0.38–1.60)
Minimum distance: 0 m	3.05 (1.30–7.11)	3.07 (1.31–7.22)	-	-
Minimum distance: AR^a^	2.96 (1.38–6.36)	2.96 (1.37–6.40)	-	-
Minimum distance: < 7 m	1.77 (0.81–3.86)	1.67 (0.76–3.65)	-	-

^a^AR means close proximity within arm’s reach

### Factors affecting the number of likes

In the results of the modeling and the AIC model selection exploring the factors that affect the number of likes of the videos (N = 206), we obtained a set of 25 candidate models (with Δ AIC values less than 6) out of 255 possible models (Table 3). Among 24 models except for the first ranked model, there were three non-nested models. The first ranked model with the minimum AIC value included five explanatory variables, the days after upload, the length of the video, the number of subscribers, the presence of silverback gorillas, the thumbnail photo type, and the minimum distance (Table 3). The number of subscribers was included in all 25 candidate models and had a positive effect on the number of likes (Tables 3 & 4). The days after upload was included in 16 candidate models and in one non-nested model. Judging from the IRR and 95% CI, the days after upload had a positive effect on the number of likes (Tables 3 & 4). A comparison of the proportion of candidate models that included the days after upload between two different response variables suggests that the variable might be having a weaker effect on the number of likes than on the number of views (Tables 1 & 3). The length of video, the population, and the presence of infant gorillas were included in 12, 11, and 11 candidate models respectively, but they were not included in any non-nested model (Tables 3 & 4). Combined with the IRR and 95% CI in the full model, we considered that these variables had almost no clear effects on the number of likes (S3 Table). The presence of silverback gorillas was included in 15 candidate models and one non-nested model (Table 3). The 95% CI lower was less than 1.00 in the first ranked model while it was more than 1.00 in the ninth ranked non-nested model (Table 4), suggesting that the presence of silverback gorillas can have a positive effect on the number of likes. The thumbnail photo type and the minimum distance had a greater effect on the number of views (Tables 3 & 4). The thumbnail photo type was included in all 25 candidate models (Table 3). Although the 95% CI lower was slightly less than 1.00 in the ninth ranked non-nested model, those of the first ranked model and the other non-nested models were more than 1.00 (Table 4). The videos with thumbnail photos that included humans and gorillas tended to obtain more likes than those with thumbnail photos that did not include gorillas (Table 4, Fig 3B). The IRR of thumbnail photos that included only gorillas were less than 1.00 in the first ranked model and the three non-nested models, but judging from the 95% CI, this variable had almost no clear effect on the number of likes compared to those with thumbnail photos that did not include gorillas (Table 4, Fig 3B). The minimum distance was included in all 25 candidate models (Table 3). Although the IRR of “< 7 m” showed a positive effect, the 95% CI included 1.00 in the first ranked model and all three non-nested models (Table 4). On the other hand, a much shorter minimum distance, namely “0 m” and “AR,” had a positive effect on the number of likes compared with videos coded as “> 7 m” in the first ranked model and all three non-nested models (Table 4, Fig 4B). A comparison of the proportion of candidate models that included the minimum distance between two different response variables suggests that the variable might be having a stronger effect on the number of likes than on the number of views (Tables 1 & 3).

**Table 3:** Top-ranked candidate models exploring the factors that affect the number of likes of YouTube videos related to mountain gorilla tourism (only the models with Δ AIC values less than 6 are shown). (N = 206).

Rank^a^	Nesting^b^	Explanatory variables								logLik	df	AIC	Δ AIC	Akaike weight
		Number of subscribes	Days after upload	Length of videos	Population	Silverback	Infant	Thumbnail	Minimum distance					
1	-	+	+			+		+	+	-967.63	10	1955.26	0.00	0.14
2	Nested	+	+	+		+		+	+	-966.98	11	1955.97	0.71	0.10
3	Nested	+	+		+	+		+	+	-967.35	11	1956.71	1.45	0.07
4	Non-nested	+	+					+	+	-969.39	9	1956.78	1.53	0.07
5	Nested	+	+	+				+	+	-968.48	10	1956.96	1.70	0.06
6	Nested	+	+			+	+	+	+	-967.59	11	1957.19	1.93	0.05
7	Nested	+	+	+	+	+		+	+	-966.69	12	1957.38	2.13	0.05
8	Nested	+	+	+		+	+	+	+	-966.93	12	1957.86	2.61	0.04
9	Non-nested	+				+		+	+	-970.04	9	1958.07	2.82	0.03
10	Nested	+	+		+			+	+	-969.24	10	1958.47	3.22	0.03
11	Nested	+	+	+	+			+	+	-968.30	11	1958.60	3.34	0.03
12	Nested	+	+		+	+	+	+	+	-967.32	12	1958.64	3.38	0.03
13	Nested	+	+				+	+	+	-969.38	10	1958.76	3.50	0.02
14	Nested	+	+	+			+	+	+	-968.48	11	1958.96	3.70	0.02
15	Nested	+		+		+		+	+	-969.53	10	1959.06	3.80	0.02
16	Nested	+			+	+		+	+	-969.62	10	1959.23	3.98	0.02
17	Nested	+	+	+	+	+	+	+	+	-966.64	13	1959.27	4.01	0.02
18	Nested	+				+	+	+	+	-969.84	10	1959.69	4.43	0.02
19	Nested	+		+	+	+		+	+	-969.08	11	1960.17	4.91	0.01
20	Nested	+	+		+		+	+	+	-969.23	11	1960.45	5.19	0.01
21	Nested	+		+		+	+	+	+	-969.29	11	1960.59	5.33	0.01
22	Nested	+	+	+	+		+	+	+	-968.30	12	1960.60	5.34	0.01
23	Non-nested	+						+	+	-972.33	8	1960.65	5.40	0.01
24	Nested	+			+	+	+	+	+	-969.42	11	1960.84	5.59	0.01
25	Nested	+		+				+	+	-971.56	9	1961.11	5.86	0.01

^a^ Rank based on Akaike information criterion (AIC) values

^b^ "Nested" indicate that the model is a more complex version of a model that has a lower AIC value

**Table 4:** Summary of the models exploring the factors that affect the number of views and likes of YouTube videos related to mountain gorilla tourism (N = 206). The incidence rate ratio (IRR) and 95% confidence interval (CI) of each explanatory variable in the first ranked model and non-nested models are shown.

Model	The first ranked model	The 4th ranked non-nested model	The 9th ranked non-nested model	The 23rd ranked non-nested model
Explanatory variables	Incidence rate ratio (IRR) (95% CI)			
Intercept	3.32 (1.14–9.53)	6.95 (3.32–14.32)	3.02 (1.03–8.70)	7.02 (3.33–14.59)
Number of subscribes	1.82 (1.42–2.33)	1.84 (1.43–2.37)	1.78 (1.39–2.29)	1.80 (1.40–2.33)
Days after upload	1.34 (1.03–1.76)	1.39 (1.07–1.81)	-	-
Length of video	-	-	-	-
Population: Virunga	-	-	-	-
Silverback: Present	2.24 (0.96–5.22)	-	2.51 (1.08–5.86)	-
Infant: Present	-	-	-	-
Thumbnail: Humans and gorillas	2.58 (1.07–6.21)	2.40 (0.99–5.83)	3.04 (1.27–7.30)	2.86 (1.18–6.93)
Thumbnail: Gorillas only	0.74 (0.37–1.47)	0.74 (0.37–1.50)	0.83 (0.42–1.66)	0.85 (0.43–1.72)
Minimum distance: 0 m	3.79 (1.68–8.59)	3.83 (1.68–8.76)	3.23 (1.44–7.29)	3.21 (1.41–7.31)
Minimum distance: AR^a^	2.76 (1.33–5.79)	2.78 (1.32–5.88)	2.37 (1.14–4.92)	2.34 (1.12–4.91)
Minimum distance: < 7 m	1.55 (0.73–3.31)	1.46 (0.68–3.14)	1.38 (0.65–2.95)	1.28 (0.59–2.74)

^a^AR means close proximity within arm’s reach

## Discussion

We have described the general characteristics of YouTube videos related to mountain gorilla tourism and analyzed the factors that affected the number of views and likes of the videos, focusing particularly on close interactions between humans and gorillas. Our results showed that among the selected 282 videos, humans and gorillas were observed simultaneously in about 70% of videos, and physical contact or close proximity within arm’s reach were identified in 40% of the videos (Fig 2). In addition, we demonstrated that in the cases where the thumbnail photos included both humans and gorillas, the videos obtained more views and likes than those that did not include gorillas, while videos with thumbnail photos that included only gorillas did not obtain more views and likes compared to those that did not include gorillas (Tables 1–4, Fig 3A and 3B). Moreover, we showed that the videos obtained more views and likes in cases where physical contact or close interaction within arm’s reach with gorillas were clearly observed compared with the videos whose minimum distances were “> 7 m” (Tables 1–4, Fig 4A and 4B). We admit that there are potential limitations associated with the representativeness of the sampled videos. We did not use the Application Program Interface (API) of YouTube to search for videos related to mountain gorilla tourism, and thus the samples might be biased. In addition, it is worth noting here that social media data are biased because they are created by the users spontaneously [40]. Therefore, we cannot say for certain whether the violation of 7 m rule is prevalent or not based on the sampled videos because of the possibility of the over-presentation. However, even with such biases, contents on social media represent about what people consider worth posting [40]. It is also generally known that the search on the API often provides many videos that are not relevant to the interest of the studies [e.g. 44, 49]. Regarding the analysis exploring factors that affect the social engagement (i.e. views and likes), we consider that the observed pattern will not be greatly affected even if we perform further analysis with more videos using the API. Therefore, it is reasonable to think that the close human-gorilla interactions in thumbnail photos and videos affect the popularity of YouTube videos related to mountain gorilla tourism.

A previous study suggested that factors that made wildlife tourism experiences memorable included the charisma of the species, spontaneity, seeing something for the first time, the degree of proximity, embodied experiences, and species congregated in large numbers [36]. Although we did not test all these factors in this study, mountain gorilla tourism seems to meet all these factors to a high degree. In particular, it is known that the degree of proximity often increases the tourists’ satisfaction in wildlife tourism experiences [e.g. 35, 36]. Our results also suggest that, in mountain gorilla tourism, human-gorilla interactions and proximity with gorillas attract more public attention than gorillas shown by themselves. It is possible that close interactions with gorillas also increase the viewers’ (and potential tourists’) satisfaction and have functioned as the main attraction.

On the other hand, it should be emphasized that proximity increases the risk of disease transmission between humans and gorillas, including respiratory disease infections as we described in the introduction. The dense vegetation and steep topography may make it difficult for observers to maintain the distance between humans and gorillas if they want to get a clear view of gorillas, especially in Bwindi [33]. In addition, some gorillas are over-habituated and often approach park rangers and tourists [33]. However, although there are not many studies in Bwindi, we must not forget that the Virunga gorilla population has experienced disease outbreaks, including human-induced ones [31] and the frequency of respiratory disease outbreaks is increasing [32]. As mentioned above, we will not make strong claims on the prevalence of the 7 m rule violation based on the videos, but previous studies suggested that the 7 m rule has not been observed strictly [33, 34].

It may be worth mentioning that preventing over-habituation is another reason for the importance of keeping the 7 m rule strictly in place [e.g. 26, 60]. Previous studies have demonstrated that several habituated gorilla groups in Bwindi range outside of the parks to feed on bananas—that may contain high sugar levels [61]—and eucalyptus trees that contain high sodium in both Bwindi and Virunga [61, 62]. It is generally considered that such behaviors increased due to the habituation [60]. Crop foraging behavior in community lands may increase the risk of disease transmission such as scabies [63] or may increase parasite loads of gorillas [e.g. 12], while it can also be a significant threat to farmers living adjacent to the parks. In addition, gorillas in Bwindi also visit tourists’ lodges where the risk of disease transmission is seemingly extremely high [26]. Indeed, in the sampled YouTube videos in this study, some human-gorilla interaction that took place in tourists’ lodges was observed.

**Fig 5:**
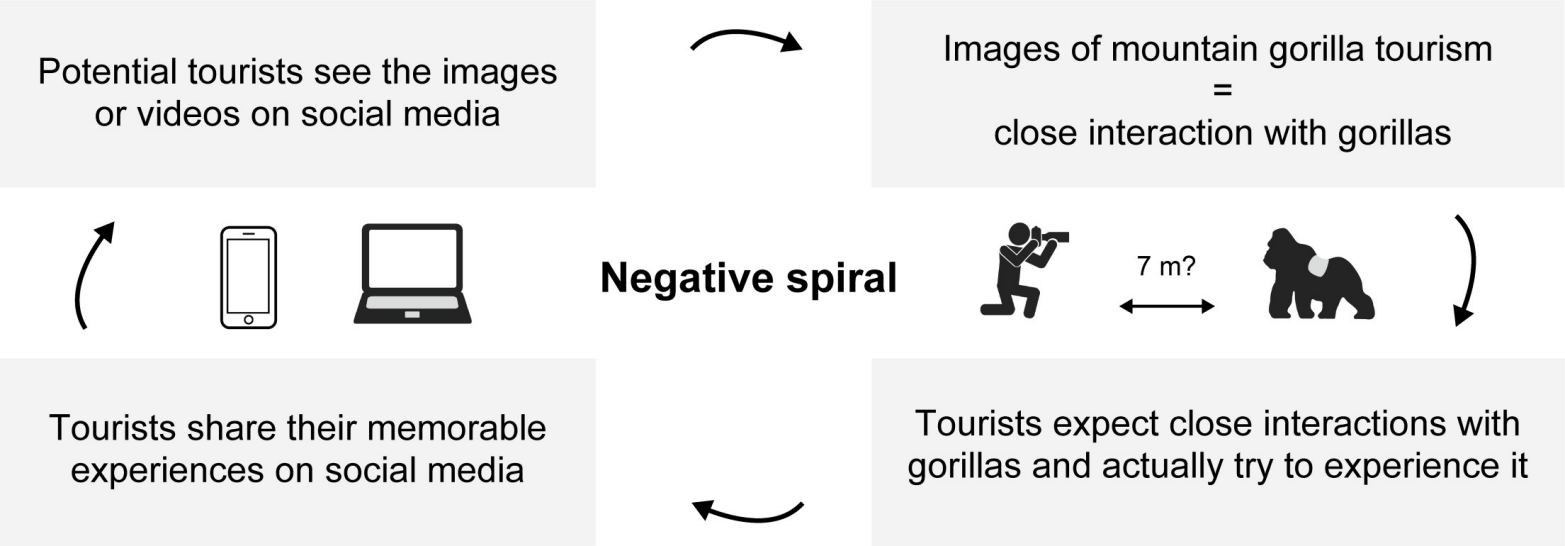
A negative spiral hypothesis regarding the violation of the regulation in mountain gorilla tourism that may be accelerated by the posting of contents that include close human-gorilla interactions on social media.

The study by Nekaris et al. 2013 [47] has highlighted that unsuitable or irresponsible content on YouTube has a powerful negative influence on public opinions for the conservation of endangered species. Previous studies, although they did not specifically focus on social media contents, have argued how media portrayals or misleading images affect public perception of wild animals [47, 64 − 66]. For instance, Ross et al. 2011 [65] showed that media portrayals of chimpanzees affect public perception on conservation of the species and the desires to own chimpanzees as a pet. Based on the literature and the result of this study, we built the following hypothesis, which we call the “negative spiral hypothesis” in mountain gorilla tourism (Fig 5). The presence of videos containing close human-gorilla interactions may drive potential tourists to expect to observe or interact with mountain gorillas from a close distance. On site, tourists who have watched such videos may try to experience what many others have experienced in the existing videos. However, the presence of such videos that clearly violate the tourism regulation makes it more difficult for both tourists and park rangers to maintain their distance. After participating in these tourism encounters, those tourists will share their memorable experiences with their relatives or friends and some of them may even share photos or videos on social media such as Facebook, Instagram, Twitter, and YouTube [e.g. 38]. Again, potential tourists who see those photos or videos will expect to experience exactly what they see. In this way, the conflicting situation is likely to be exacerbated in mountain gorilla tourism (Fig 5).

We would like to emphasize here that this hypothesis should be discussed further in a broader context. For instance, there might be an “innate” desire to interact with nature/wildlife on the human side [67]. In this context, the results and hypothesis suggest that such desires may drive people living far from the natural environment to search for, watch, and like social media contents that include human-wildlife close interactions. It may also drive potential tourists to expect close interactions with wildlife or such social media contents may stimulate and enhance such expectations or desires. On the other hand, it is also possible that such tourist expectations or desires have been constructed and reinforced in complex social contexts through popular media. In popular media, there have been some potentially misleading photos and footages that illustrated close interaction (including physical contact) between great apes and famous people such as scientists, conservationists, photographers, filmmakers, talents and so forth. In this context, although the impact of such popular media on public perceptions have been powerful, our hypothesis highlights that the impact of social media contents that are still mainly generated by “non-famous”, “non-professional” people have also become or may become more powerful in this Information Age.

We also admit that our analysis alone may not fully explain the direct relation between the popularity of YouTube contents that illustrate human-gorilla close interaction and the chances of population decline. Yet, it must be worth mentioning here that veterinarians sometimes intervene and treat the animals if they show disease symptoms [25, 30 − 32] and it might be difficult to simply associate the probability of the disease transmission with population dynamics. In any case, however, further studies are required to test the hypothesis and demonstrate how and how much such contents on social media affect viewers’ (or potential tourists’) perceptions and behavior and how much these perceptions and behavior affect the probability of disease transmission.

From a practical point of view, it is recommended that all actors related to mountain gorilla tourism, including park rangers and officers, porters, lodge staff, NGO staff, researchers, influential media, tour operators, and tourists must always be aware of the potential negative effects of close proximity to mountain gorillas [e.g. 10, 29 − 32]. Tourists must be properly informed about the risk of disease transmission and other negative effects before, during, and after observing gorillas. Tourists and park rangers should always try their best to maintain their distance, resisting the temptation to observe, interact, and take photos or videos at close proximity to the gorillas. It is necessary to organize frequent training workshops for park rangers to increase their performance in managing the distances between tourists and gorillas [33, 34] while maintaining the tourists’ satisfaction. It might be effective to control the number of tourists in order to enhance the management of tourism regulations and increase tourists’ satisfaction [e.g. 4, 26]. In addition, a possible measure to minimize the negative effects of ecotourism, especially for respiratory diseases, is the use of surgical masks [e.g. 10, 26, 29, 34]. Although it has been proposed for a long time, the use of masks has not yet been implemented except in Virunga National Park in the Democratic Republic of Congo. The use of surgical masks should be mandatory for anyone who tracks gorillas, aiming not to justify close interactions but to raise awareness of the risk and to prevent droplet infections [26].

On social media, the uploaders of contents that violate tourism regulations are required to be aware of the potential negative effects of such contents on public perception [e.g. 47, 65] or potential tourists’ perception and behavior. It may not be justified even if the uploaders intended to contribute to conservation by advertising ecotourism or relating the conservation status of the species. On the other hand, the viewers are required to interpret such contents on social media carefully. We have discussed the potential negative effects, but at the same time, we consider that such contents can also provide educational opportunities if they can stimulate discussion about the appropriate distance between humans and great apes during observation in their natural habitats. It is extremely important to deliver correct information on social media about the conservation statuses of endangered species [47, 68] and social media has the potential to provide positive outcomes in conservation [e.g. 69]. In the case of mountain gorilla tourism, much effort is needed both on site and on social media in order to increase public awareness and knowledge regarding the negative effects of close human-gorilla interaction during mountain gorilla tourism experiences.

## Supporting information

S1 Table The explanatory variables we used in models exploring factors that affect the number of views and likes of YouTube videos related to mountain gorilla tourism. (DOCX)

S2 Table Summary of the full model exploring factors that affect the number of views of YouTube videos related to mountain gorilla tourism. (N = 206).
(DOCX)

S3 Table Summary of the full model exploring factors that affect the number of likes of YouTube videos related to mountain gorilla tourism. (N = 206).
(DOCX)

S1 Dataset Dataset for PLOS ONE. (XLSX)
