# Supplementary material for: Analyzing the popularity of YouTube videos that violate mountain gorilla tourism regulations
Source: PLoS One. 2020 May 21;15(5):e0232085. doi: 10.1371/journal.pone.0232085 (PMC7241773; doi:10.1371/journal.pone.0232085)
Supplement: S1 Table — (DOCX) [file pone.0232085.s001.docx]

**S1 Table. The explanatory variables we used in models exploring factors that affect the number of views and likes of YouTube videos related to mountain gorilla tourism. (N=206)**

| Explanatory variables |  | N |
| --- | --- | --- |
| Number of subscribers | Continuous variable | 206 |
| Days after upload | Continuous variable | 206 |
| Length of video | Continuous variable | 206 |
| Population | Bwindi | 101 |
|  | Virunga | 105 |
| Silverback | Absent | 22 |
|  | Present | 184 |
| Infant | Absent | 37 |
|  | Present | 169 |
| Thumbnail photo type | Others | 39 |
|  | Humans and gorillas | 42 |
|  | Gorillas only | 125 |
| Minimum distance | > 7 m | 46 |
|  | < 7 m | 51 |
|  | AR | 61 |
|  | 0 m | 48 |
