# Supplementary material for: Analyzing the popularity of YouTube videos that violate mountain gorilla tourism regulations
Source: PLoS One. 2020 May 21;15(5):e0232085. doi: 10.1371/journal.pone.0232085 (PMC7241773; doi:10.1371/journal.pone.0232085)
Supplement: S2 Table — (N = 206). (DOCX) [file pone.0232085.s002.docx]

**S2 Table. Summary of the full model exploring factors that affect the number of views of YouTube videos related to mountain gorilla tourism. (N=206)**

| Explanatory variables | Incidence rate ratio (IRR) | 95% CI | | Estimate | SE | z value | p value |
| --- | --- | --- | --- | --- | --- | --- | --- |
|  |  | lower | upper |  |  |  |  |
| Intercept | 588.74 | 174.99 | 1980.68 | 6.38 | 0.62 | 10.35 | <0.001 |
| Number of subscribes | 1.78 | 1.36 | 2.32 | 0.57 | 0.14 | 4.20 | <0.001 |
| Days after upload | 2.74 | 2.07 | 3.62 | 1.01 | 0.14 | 7.10 | <0.001 |
| Length of video | 1.05 | 0.80 | 1.38 | 0.05 | 0.14 | 0.39 | 0.700 |
| Population: Virunga | 1.05 | 0.61 | 1.81 | 0.05 | 0.28 | 0.18 | 0.861 |
| Silverback: Present | 2.29 | 0.94 | 5.58 | 0.83 | 0.45 | 1.82 | 0.068 |
| Infant: Present | 0.83 | 0.40 | 1.73 | -0.19 | 0.37 | -0.51 | 0.613 |
| Thumbnail: Humans and gorillas | 2.59 | 1.02 | 6.59 | 0.95 | 0.47 | 2.01 | 0.045 |
| Thumbnail: Gorillas only | 0.67 | 0.33 | 1.36 | -0.40 | 0.36 | -1.11 | 0.266 |
| Minimum distance: 0 m | 3.02 | 1.29 | 7.06 | 1.10 | 0.43 | 2.55 | 0.011 |
| Minimum distance: AR^a^ | 2.89 | 1.31 | 6.34 | 1.06 | 0.40 | 2.65 | 0.008 |
| Minimum distance: < 7 m | 1.71 | 0.78 | 3.76 | 0.54 | 0.40 | 1.35 | 0.179 |

**^a^AR means close proximity within arm’s reach**
