# Supplementary material for: Analyzing the popularity of YouTube videos that violate mountain gorilla tourism regulations
Source: PLoS One. 2020 May 21;15(5):e0232085. doi: 10.1371/journal.pone.0232085 (PMC7241773; doi:10.1371/journal.pone.0232085)
Supplement: S3 Table — (N = 206). (DOCX) [file pone.0232085.s003.docx]

**S3 Table.** **Summary of the full model exploring factors that affect the number of likes of YouTube videos related to mountain gorilla tourism. (N=206)**

| Explanatory variables | Incidence rate ratio (IRR) | 95% CI | | Estimate | SE | z value | p value |
| --- | --- | --- | --- | --- | --- | --- | --- |
|  |  | lower | upper |  |  |  |  |
| Intercept | 3.35 | 1.05 | 10.67 | 1.21 | 0.59 | 2.05 | 0.041 |
| Number of subscribes | 1.82 | 1.42 | 2.33 | 0.60 | 0.13 | 4.72 | <0.001 |
| Days after upload | 1.33 | 1.02 | 1.74 | 0.29 | 0.14 | 2.12 | 0.034 |
| Length of video | 1.17 | 0.90 | 1.51 | 0.15 | 0.13 | 1.17 | 0.241 |
| Population: Virunga | 1.23 | 0.73 | 2.06 | 0.21 | 0.26 | 0.78 | 0.438 |
| Silverback: Present | 2.24 | 0.95 | 5.28 | 0.81 | 0.44 | 1.85 | 0.065 |
| Infant: Present | 0.89 | 0.44 | 1.79 | -0.12 | 0.36 | -0.34 | 0.736 |
| Thumbnail: Humans and gorillas | 2.75 | 1.13 | 6.69 | -0.26 | 0.35 | -0.73 | 0.463 |
| Thumbnail: Gorillas only | 0.77 | 0.39 | 1.53 | 1.01 | 0.45 | 2.24 | 0.025 |
| Minimum distance: 0 m | 3.61 | 1.61 | 8.09 | 1.28 | 0.41 | 3.12 | 0.002 |
| Minimum distance: AR^a^ | 2.49 | 1.18 | 5.27 | 0.91 | 0.38 | 2.39 | 0.017 |
| Minimum distance: < 7 m | 1.45 | 0.69 | 3.09 | 0.37 | 0.38 | 0.98 | 0.329 |

^a^AR means close proximity within arm’s reach

Please note that the 95% CI of estimates in this full model was calculated approximately by setting the method to “Wald” in the confint function, while those of other models presented in the article and supporting information were “profile” confidence intervals.
